# The Duty of the Practitioner in Acute Intestinal Obstruction

**Published:** 1900-11-24

**Authors:** 


### THE DUTY OF THE PRACTITIONER IN ACUTE INTESTINAL OBSTRUCTION.

Now, if we turn from this side of the question and consider what operation should be done, where it should be done, and by whom, Dr. Maylard has much to say, which should be carefully considered by general practitioners, and especially by those who are far removed from hospitals and consultants.

What we have constantly to bear in mind is that time is the first element in success; that the initial symptoms in cases of acute obstruction are quickly followed by others of a toxic nature, which introduce complications difficult to overcome, and that it is often far better for the patient to have an emergency operation done—even by one who may not be constantly engaged in operative work—than to wait any considerable time for the best of surgeons or the most complete of operations. As to the question, what operation should be done, Dr. Maylard says: "Were all men operating surgeons in the sense of being hospital surgeons, or did all practitioners live in close proximity to a properly equipped nursing home, the question would not be such a difficult one to answer." But the difficulties are great with men unaccustomed to operative work of the kind involved in such cases, and unprovided with the facilities of the most needed kind; and we think that Dr. Maylard has done good service in pointing out how simple may be the operative measures which may be sufficient to tide over the emergency, always supposing that they are undertaken during the stage of the initial symptoms and before the case has been complicated by the onset of that secondary toxæmia which is the actual cause of death in so many cases.

Peritonism arising from perforation is another matter, the finding and the closing of an orifice in one of the hollow viscera being an affair which will try anyone's resources, especially if he has not proper appliances and assistance. But when one can feel sure that one has to do with a case of acute obstruction following upon a history of chronic obstructive trouble—a condition which is not usually difficult to diagnose—the opening of the colon in the left lumbo-iliac region when the obstruction is known to be in the rectum, and in the right lumbo-iliac region when it is thought to be somewhere between the rectum and the cæcum, is not a great affair; while in the case of acute obstruction arising from undetermined cause "the duty of the practitioner is equally clear and equally simple. The old classical operation of Nelaton is the proper one to perform. . . . The operation simply consists in making an oblique incision about a couple of inches long through the abdominal parietes in the right iliac or inguinal region. The distended coil of intestine which presents is carefully secured by stitches to the edges of the parietal wound and the bowel opened by a small incision." If by doing either of these operations the practitioner succeeds in saving his patient's life he may be well content. As for what is to be done with the case afterwards, that is another matter. Time is gained, and if the emergency operation do not actually cure, which sometimes it does in cases of acute obstruction, the patient is at least put into such a position that he can safely wait for more special surgical assistance.

Dr. Maylard says, "No medical man . . . however isolated his position, or devoid he is of the modern requirements of surgical practice, should hesitate to give his patient that chance of life which such a simple and comparatively safe operation affords." But, we would add, let not the practitioner imagine that a case of obstruction may be allowed to run on, and then, after toxic mischief has been set up, may be brought round by such simple measures. No, if we read Dr. Maylard aright, his aim in urging the general practitioner to operate is that the patient may get the benefit of operation within that short limit of time during which operation can be undertaken with the greatest prospect of success.

